# Mandible Reconstruction

**Published:** 2013-02-01

**Authors:** Jennifer E. Kim, Justin M. Broyles, Justin M Sacks

**Affiliations:** Department of Plastic and Reconstructive Surgery, The Johns Hopkins University School of Medicine, Baltimore, Md

**Figure:**
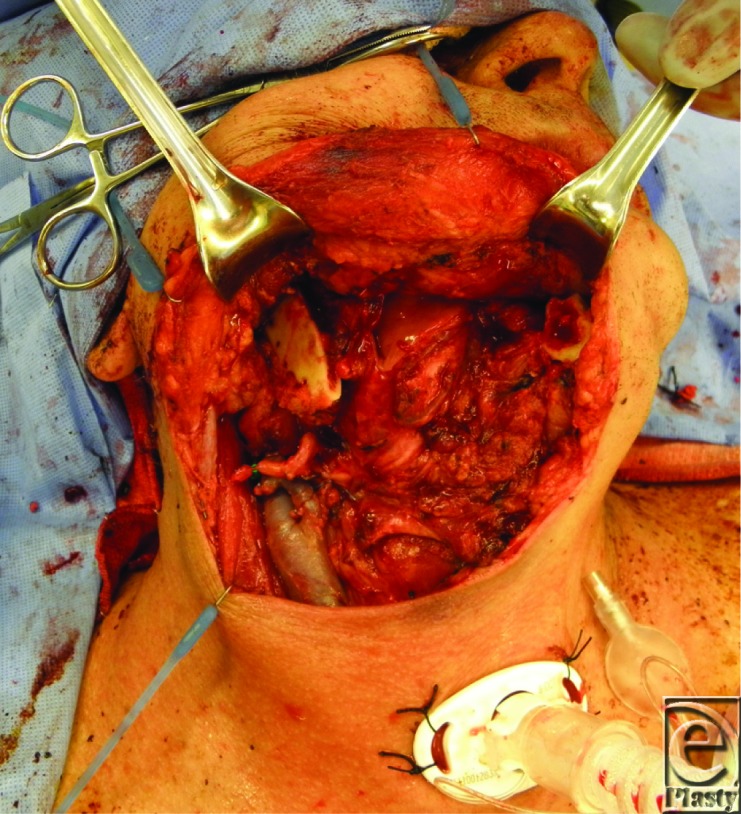


## DESCRIPTION

A 62-year-old man presents with a 5-cm anterior defect of the mandible extending from the parasymphyseal region to the angle after radiation and oncologic resection. A free fibular flap is utilized to reconstruct both soft tissue and bony anatomy.

## QUESTIONS

1. **What are the advantages of and indications for using a free fibular flap in mandibular reconstruction?**
2. **What are the expected outcomes and complications?**
3. **Are there any inherent challenges or limitations in working with the free fibular flap?**
4. **What steps should be taken in the preoperative planning phase?**

## DISCUSSION

Oncologic resection secondary to epidermoid carcinoma or osteogenic sarcoma is the leading cause of segmental mandible defects. Benign cystic or fibrotic bone diseases can also lead to segmental loss, and gunshot wounds represent the majority of traumatic causes. These defects are generally classified according to bone loss. Characterizing the extent of soft tissue defects is also important, because they have been shown to better correlate with functional and esthetic outcomes than bony defects alone.^1^ Any one or combination of these can lead to significant functional, communicative, and esthetic impairments. Reconstruction is warranted to reestablish the tandem temporomandibular joint action and facial symmetry.

The options for mandibular reconstruction include nonvascularized bone grafts, metal plates, and pedicled or free flaps. Of these, the free fibular flap (FFF) has become the gold standard due to its unmatched ability to accommodate varying degrees of resection. The FFF provides straight, sturdy, uniformly thick, and sufficiently lengthy (22-26 cm, adult) bone to reconstruct angle-to-angle defects. The bone receives a segmental supply of periosteal blood, accommodating a wide range of osteotomy sites and its bicorticocancellous nature allo ws for future osseointegrated dental implants. The flap also provides a 2- to 3-mm diameter pedicle of up to 15 cm in length and a thin, pliable skin paddle of up to 25 × 14 cm^2^ that can be taken with the underlying muscle to fill dead space or oral soft tissue defects. The sural nerve may also be harvested simultaneously in cases where reconstruction of the inferior alveolar nerve is needed. Additional benefits include the donor site location which allows for 2 teams to work concurrently and acceptable donor site morbidity.^2^ In general, the FFF is the primary choice for reconstructing anterior mandibular defects, because the skin island can be rotated across to cover the floor of the mouth and the flexor hallucis longus muscle used to fill space around the mandible and upper neck. For lateral defects, the ilium, radius, scapula, or fibula may be chosen depending on the degree of resection.^3^ Other methods of reconstruction have been used with varying degrees of success, and with significant caveats. Metal plates and prosthetics require soft tissue coverage, but the combination has been associated with unacceptable rates of flap failure.^4^ Pedicled trapezius and pectoralis osteomyocutaneous flaps often do not provide enough tissue. Iliac crest flaps have limited sculptability due to the bone's inherent shape. The radius tends to fracture under load and is easily devascularized.

Retrospective outcomes research gives strong evidence that FFF mandible reconstruction yields the most favorable functional and esthetic results with relatively low rates of complication and donor-site morbidity. A 10-year follow-up study by Hidalgo et al demonstrated a 100% flap survival among 20 patients with FFF reconstruction after oncologic resection, with 13 patients tolerating a regular diet and 16 able to speak intelligibly. Excellent esthetic outcomes were seen in 53% of the patients, good in 21%, fair in 16%, and poor in 11%; asymmetry was only slightly accentuated during natural facial aging.^1^

A technical challenge is the limited amount of soft tissue that can be harvested from the FFF donor site. Because of the thinness of the myocutaneous portion, there is an increased risk of leaving dead space when the FFF is used as a single composite flap. This dead space is associated with total flap necrosis and postoperative thrombosis secondary to infection. Thus, in cases of extensive soft tissue resection or a through-and-through mandibulectomy, the FFF may be supplemented with a forearm, anterior thigh, or rectus abdominus flap for added bulk. The use of a double-skin paddle FFF has also been proposed as a safe and reliable way to reconstruct through-and-through anterior resections with large cutaneous defects.^2^^,^^5^

Despite its limitations, the main advantage of the FFF is its unmatched sculptability. The straight bone lends itself to the replacement of lateral segments, or can be recontoured in cases of central defects. However, the process of fabricating a 3-dimensional anterior mandibular arch from linear bone is complex and requires preoperative calculation to predict how much bone and soft tissue will need be harvested during the procedure. Precise cuts and apposition of the fibular graft and native mandible are required to achieve maximal alveolar alignment and satisfactory cosmesis with respect to the external contour of the lower jaw. Traditionally, decisions on how bone is to be reshaped are made intraoperatively, guided by silastic sheet templates based on stenciled margins of the resected specimen. In recent years, however, there has been greater interest computer-assisted surgical modeling and template design as a means of minimizing technical difficulties and maximizing functional and esthetic outcomes. Stereolithography can be used to collect data on the 3-dimensional orientation and size of the proposed defect, while angiographic magnetic resonance or angiographic computed tomography facilitates preoperative evaluation of the length, caliber, and patency of the pedicle. Both imaging studies can help minimize the intraoperative time needed to measure out the exact amount of tissue to be harvested and to precisely shape and approximate the neomandible to the original structure. This also minimizes the risk of ischemic injury during flap preparation or microvascular anastomosis.^1^^,^^3^
